# Efficient Recovery of Valeric Acid Using Phosphonium-Based Ionic Liquids

**DOI:** 10.3390/ijms26188970

**Published:** 2025-09-15

**Authors:** Alexandra Cristina Blaga, Oana Cristina Parvulescu, Dan Cascaval, Anca Irina Galaction

**Affiliations:** 1 “Cristofor Simionescu” Faculty of Chemical Engineering and Environmental Protection, “Gheorghe Asachi” Technical University of Iasi, 67 Dimitrie Mangeron Av., 700050 Iasi, Romania; dan.cascaval@academic.tuiasi.ro; 2 Faculty of Chemical Engineering and Biotechnologies, National University of Science and Technology POLITEHNICA Bucharest, 1-7 Gheorghe Polizu St., 011061 Bucharest, Romania; 3 Faculty of Medical Bioengineering, “Grigore T. Popa” University of Medicine and Pharmacy, 9-13 Mihail Kogalniceanu St., 700454 Iasi, Romania; anca.galaction@umfiasi.ro

**Keywords:** heptane, ionic liquid, reactive extraction, valeric acid

## Abstract

This study explores the application of phosphonium-based ionic liquids (ILs) for the efficient separation of valeric acid (VA) through reactive liquid–liquid extraction. Two hydrophobic quaternary phosphine ILs, trihexyl(tetradecyl)phosphonium decanoate (C103) and trihexyl(tetradecyl)phosphonium bis(2,4,4-trimethylpentyl)phosphinate (C104), were evaluated in combination with heptane as a diluent. Extraction efficiency was experimentally determined at different levels of extraction process factors in terms of aqueous phase pH (3–6), IL concentration (0–120 g/L), and process temperature (25–60 °C). For each IL, extraction efficiency was predicted using a response surface regression model, and the process factors were optimized based on the desirability function approach. Both ILs effectively extracted VA, with optimal mean values of extraction efficiency of 98.61% for C103 and 99.24% for C104 under optimal conditions (pH of 3.8 and 4, respectively, IL concentration of 60 g/L, and temperature of 25 °C). Mechanistic analysis revealed that VA extraction occurs through the formation of IL-acid complexes, involving hydrogen bonding between the non-dissociated acid and the IL anion. Depending on the extractant concentration, 1:1 and 2:1 acid-to-IL stoichiometric ratios were observed. These findings highlight the potential of phosphonium-based ILs, particularly in a heptane-diluted system, as high-performance extractants for carboxylic acid separation.

## 1. Introduction

Volatile fatty acids (VFAs) are essential chemical intermediates with diverse applications across industries such as food, pharmaceuticals, plastics, and wastewater treatment. These low-molecular-weight organic acids can be derived both from petrochemical processes and the bioconversion of organic matter. VFAs are important by-products of fermentation and digestion processes, primarily consisting of six short-chain fatty acids: acetic acid, propionic acid, *n*- and *i*-butyric acid, and *n*- and *i*-valeric acid [1]. In addition to serving as a supplementary carbon source for nitrogen and phosphorus removal in sewage treatment [2,3], VFAs are also valuable raw materials for producing biodegradable plastics such as polyhydroxyalkanoates [4,5]. The fermentative production of VFAs from waste activated sludge (WAS) involves a critical balance between hydrolysis, acidification, and methanation, with hydrolysis being the rate-limiting step [6,7]. Various pretreatment methods, including alkaline treatment, ultrasonication, microwave heating, and the addition of free nitrous acid, can enhance the hydrolysis rate and promote the production of fermentation intermediates [8]. However, these methods tend to be energy-intensive and may face challenges from emerging pollutants in WAS, which limits their large-scale application in wastewater treatment plants [6,9].

Among VFAs, valeric acid (VA), also known as pentanoic acid (C_5_H_10_O_2_), stands out for its considerable industrial potential. VA is a straight-chain saturated carboxylic acid with a strong, unpleasant odor, naturally occurring in both free and esterified forms in various plants, particularly those of the *Valeriana* genus within the *Valerianaceae* family [10]. Despite its pungent smell, VA possesses a distinctive fruity aroma that makes it a valuable intermediate in the fragrance, cosmetics, pharmaceutical, and food industries [11]. It also plays an important role in various bioproduction processes, including the manufacture of monoclonal antibodies, animal feed, flavorings, pharmaceutical compounds, lubricants, and biodegradable plastics [6].

VA can be obtained from plants like *Angelica archangelica* and *Valeriana officinalis*, produced through the oxidation of biosynthesized amyl alcohol [9], or via biosynthesis from renewable resources. One efficient route involves coupling two fermentation processes: the initial production of propionic acid followed by elongation chain to valeric acid [12]. Additionally, the selective reduction in levulinic acid to valeric acid represents a key reaction in converting biomass and its derivatives into fuel precursors. This transformation significantly contributes to the advancement of bio-based production systems, with promising potential to reduce fossil fuel dependency and address pressing environmental concerns [13]. The production of VA from WAS through fermentative pathways has emerged as a promising strategy for sustainable industrial applications that not only enables the efficient valorization of residual materials but also contributes to CO_2_ emissions reduction and supports the transition toward a circular and sustainable economy [3,6,14]. This approach transforms waste into a valuable resource with applications in bioenergy and biodegradable materials. Table 1 presents reported values of VA production achieved through waste sludge fermentation.

The growing demand for environmentally friendly and cost-effective separation techniques has led to increased interest in alternative methods for VA recovery and purification. Liquid–liquid reactive extraction, a common separation method, is advantageous for its efficiency in separating VFAs, especially from low-concentration fermentation broths. Several studies have demonstrated the use of various extractants and solvents for the recovery of VFAs. For example, in case of VFA separation from mixed culture fermentation broth, according to Kaur et al. (2020), in situ product recovery using reactive extraction with extractants such as Aliquat 336 and trioctylphosphine oxide (TOPO) in methyl octanoate achieved 75–85% higher extraction efficiency than physical extraction [19]. Similarly, Alkaya et al. (2009) used TOPO in kerosene for the extraction of various VFAs, including acetic, propionic, butyric, iso-butyric, valeric, iso-valeric, caproic, iso-caproic, and heptanoic acids [20]. The extraction was conducted on 10 mL of fermentation broth, which was derived from the simultaneous acidification of sugar industry wastewater and beet pulp, resulting in VFA concentrations of 2910 mg/L, and up to 98% VFA recovery. Furthermore, Rocha et al. (2017) demonstrated up to 80% extraction efficiency of acetic, propionic, and butyric acids using medium-chain fatty acids (hexanoic, octanoic, and decanoic acids) diluted in organic solvents such as toluene and *n*-hexane, with an initial VFA concentration of 1 wt% [21]. Begun et al. (2020) demonstrated that reactive extraction of VFAs using extractants such as tri-*n*-octylamine (TOA) and tributyl phosphate (TBP) in 1-octanol as a diluent achieved 90.9% in synthetic systems, but the system had a reduced performance in real effluents due to matrix complexity and co-extraction effects [22].

Ionic liquids (ILs) have emerged as a promising green alternative for the extraction and separation of VFAs due to their unique physicochemical properties, including low volatility, controllable solubility, and high thermal stability. Reyhanitash et al. (2019) investigated the VFA extraction efficiency of various solvents and found that ILs, particularly trihexyl(tetradecyl)phosphonium bis-2,4,4-(trimethylpentyl) phosphinate ([P_66614_][Phos]), were highly effective in extracting lactic acid, acetic acid, propionic acid, and butyric acid. Using a 3:5 IL to VFA volume ratio, they achieved a VFA extraction yield of 100 mg/g IL [23]. Singh et al. (2024) further explored the hydrophobic IL [P_66614_][Dibutylphosphate] for extracting a wider range of VFAs, including acetic, propionic, butyric, and hexanoic acids [24]. Their results demonstrated the superior extraction capacity of this IL, achieving a remarkable yield of 842.8 mg/g IL. Andersen et al. (2016) also studied ILs for VFA recovery, specifically [P_66614_][Dicyanamide], and achieved a VFA extraction yield of 48.4 mg/g IL at a 1:10 IL to VFA volume ratio [25]. In a similar approach, Xing et al. (2023) examined [P_66614_][Chloride] for extracting acetic acid and butyric acid [26]. They reported extraction yields of 190–250 mg/g IL for acetic acid and 330–460 mg/g IL for butyric acid, using a 2:1 IL to VFA volume ratio with 20% IL in dodecane. These studies highlight the potential of ILs as highly effective and versatile solvents for VFA extraction, with varying extraction efficiencies depending on the type of IL, VFA, and extraction conditions used.

This study investigated the potential of ILs for the separation of VA from aqueous solutions using two types of quaternary phosphine ILs, trihexyl-tetra-decyl-phosphonium decanoate (C103) and trihexyl-tetra-decyl-phosphonium bis(2,4,4-trimethylpentyl)phosphinate (C104), in combination with the green solvent heptane. This study uniquely integrates trihexyl-tetradecylphosphonium-based ILs with heptane as a green diluent, addressing the critical challenges of IL viscosity and phase separation in nonpolar solvents, while systematically investigating the combined effects of aqueous phase pH, extractant concentration, and temperature on valeric acid extraction efficiency. This comprehensive approach not only advances the understanding of IL–diluent interactions but also demonstrates a scalable and effective reactive extraction method for valeric acid, which has not been extensively explored in prior literature. To evaluate the influence of process variables and extractant type on extraction efficiency, statistical analysis, including one-way ANOVA and *t*-test for two samples with equal and unequal variances, and response surface regression models were used.

## 2. Results

### 2.1. Experimental Results

Reactive liquid–liquid extraction is an innovative and efficient technique used to improve the performance of fermentative production and downstream processing of bio-based organic acids by transferring them into an organic phase. This study aims at improving the extraction yield for the effective recovery of VA from dilute aqueous solutions using an IL as the extractant, combined with the low-polarity solvent heptane as the diluent. Key factors influencing the reactive extraction process include aqueous phase pH (*pH*), IL concentration (*IL*), and extraction temperature (*t*).

**Figure 1:**
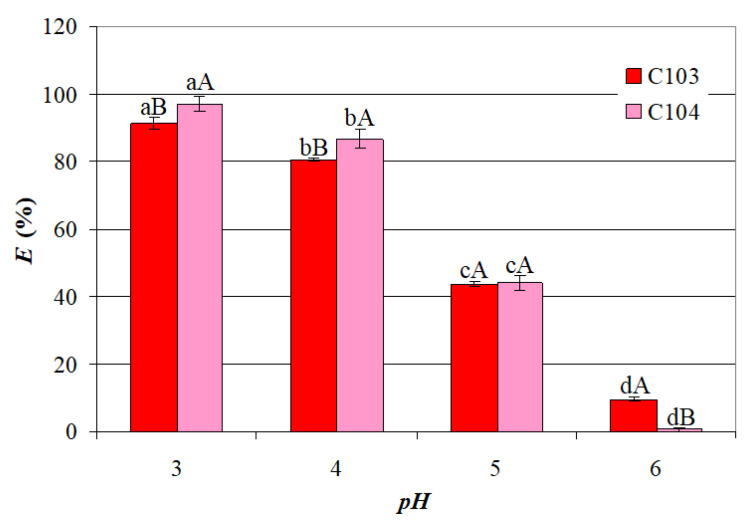
Aqueous phase pH influence on VA extraction efficiency (*IL* = 40 g/L, *t* = 25 °C); different lowercase letters indicate significant differences (*p* < 0.05) between *E* obtained at different *pH* values for each IL; different uppercase letters indicate significant differences (*p* < 0.05) between *E_C_*_103_ and *E_C_*_104_ obtained at a certain *pH* value.

Extensive research has demonstrated that *pH* plays a critical role in the extraction of carboxylic acids by controlling the equilibrium between the aqueous and organic phases [27]. Carboxylic acids are typically extracted more efficiently at low *pH* values, where they exist in their non-dissociated form, usually below the *pK_a_* of the acid. The extraction efficiency (*E*) of the extractants (C103 and C104) on VA at *IL* = 40 g/L, *t* = 25 °C, and different *pH* levels (3–6) is shown in Figure 1. One-way ANOVA applied to the data presented in Figure 1 highlighted the following factors:

1. A significant decrease in the mean values of *E* with an increase in *pH* from 3 to 6, i.e., from 91.21% to 9.489% for C103 and from 96.95% to 0.694% for C104;
2. Significantly higher mean values of *E_C_*_104_ than those of *E_C_*_103_ for *pH* = 3 and *pH* = 4, similar mean values of *E_C_*_103_ and *E_C_*_104_ for *pH* = 5, and significantly higher mean value of *E_C_*_103_ than that of *E_C_*_104_ for *pH* = 6.

These findings indicate that the highest *E* values were attained when VA exists primarily in its molecular form, facilitating effective separation when the *pH* is below its *pK_a_* (4.84 [28]).

**Figure 2:**
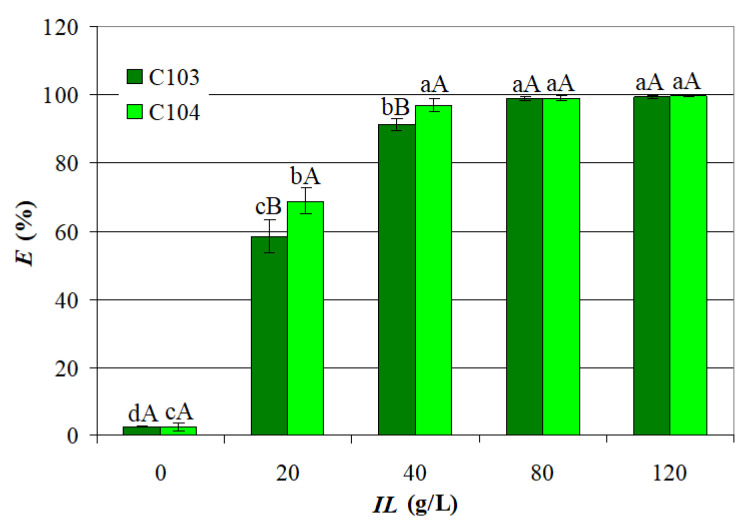
ILs concentration influence on VA extraction efficiency (*pH* = 3, *t* = 25 °C); different lowercase letters indicate significant differences (*p* < 0.05) between *E* obtained at different *IL* values for each IL; different uppercase letters indicate significant differences (*p* < 0.05) between *E_C_*_103_ and *E_C_*_104_ obtained at a certain *IL* value.

The choice of IL and solvent combination in the organic phase plays a vital role in optimizing carboxylic acid extraction. ILs are considered effective and environmentally friendly extractants [29], and their combination with suitable diluents, such as heptane, can further enhance extraction performance by reducing viscosity and improving mass transfer. A comprehensive investigation into the mechanism and stoichiometry of VA–ILs complex formation requires evaluating the impact of ILs concentration on extraction performance. The influence of *IL* (0–120 g/L) on *E_C_*_103_ and *E_C_*_104_ at *pH* = 3 and *t* = 25 °C is shown in Figure 2. Depicted data highlight a sharp increase in the mean values of *E_C_*_103_ and *E_C_*_104_ with an increase in *IL* from 0 to 40 g/L (0.061 and 0.051 mol/L for C103 and C104, respectively), i.e., from 2.565% to 91.21% for C103 and from 2.565% to 96.95% for C104. For *IL* = 20 g/L and *IL* = 40 g/L, the mean values of *E_C_*_104_ (68.86% and 96.95%) were significantly higher (*p* ≤ 0.05) than those of *E_C_*_103_ (58.57% and 91.21%). Compared with the results obtained in the absence of ILs, this improvement in *E* can be attributed to specific chemical interactions [23] between the ILs and the acid molecules, involving the formation of acid–IL complexes in the organic phase, which facilitates the transfer of VA from the aqueous phase. However, at higher levels of *IL*, i.e., 80 g/L and 120 g/L, the mean values of *E_C_*_103_ and *E_C_*_104_ (98.83–99.59%) were not significantly different (*p* > 0.05), suggesting a saturation effect of the extractants.

**Figure 3:**
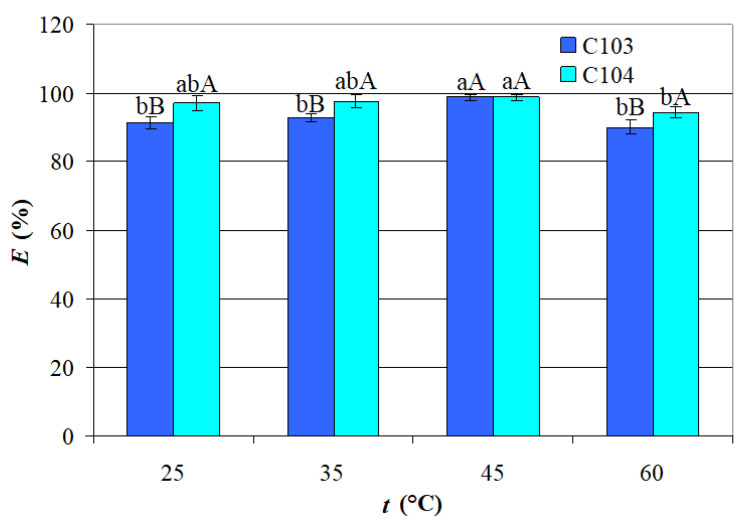
Temperature influence on VA extraction efficiency (*pH* = 3, *IL* = 40 g/L); different lowercase letters indicate significant differences (*p* < 0.05) between *E* obtained at different *t* values for each IL; different uppercase letters indicate significant differences (*p* < 0.05) between *E_C_*_103_ and *E_C_*_104_ obtained at a certain *t* value.

Temperature plays a critical role in the reactive extraction of carboxylic acids, including VA, particularly due to its impact on both the extraction and back-extraction (regeneration) processes. As reactive extraction systems typically operate within specific temperature ranges, understanding how temperature influences extraction efficiency is essential for optimizing process conditions. The influence of *t* (25–60 °C) on *E_C_*_103_ and *E_C_*_104_ at *pH* = 3 and *IL* = 40 g/L is shown in Figure 3.

Depicted data indicate the following

1. The mean value of *E_C_*_103_ obtained at *t* = 45 °C (98.59%) was significantly higher than the mean values of *E_C_*_103_ attained at the other *t* levels (90.05–92.89%), which were not significantly different; this trend may be attributed to the system approaching equilibrium conditions, where complexation between the acid and ILs occurs predominantly at the organic-aqueous interface; these complexation reactions are generally exothermic and sensitive to thermal variations; as temperature increases, the kinetic energy of molecules also rises, potentially disrupting weaker interactions while promoting the formation of more stable acid–IL ILs complexes; additionally, the exothermic nature of hydrogen bonding involved in complex formation may contribute to a reduction in system entropy, further influencing extraction behavior [30].
2. The mean value of *E_C_*_104_ obtained at *t* = 45 °C (98.68%) was significantly higher than that attained at *t* = 60 °C (94.23%);
3. The mean values of *E_C_*_103_ and *E_C_*_104_ reached at *t* = 45 °C were similar, whereas the mean values of *E_C_*_104_ obtained at the other *t* levels (94.23–97.49%) were significantly higher than those of *E_C_*_103_ (90.05–92.89%).

The lower aqueous solubility of both ILs (C103: 8.7 ± 0.2 mg/L, C104: 12.6 ± 0.3 mg/L [31]) suggests a reduced risk of IL loss during back-extraction, which is essential for maintaining regeneration efficiency and long-term reusability (critical factors for industrial implementation). Back-extraction of valeric acid (VA) was carried out using a sodium hydroxide (NaOH) solution (pH 12), which effectively re-extracted the acid due to its strong basicity. Performing the regeneration step at 45 °C further enhanced back-extraction efficiency by shifting the extraction equilibrium in favor of VA release from the IL-acid complex. Although the re-extraction efficiency was 73.46%, the ILs retained high extraction capacity in optimum conditions over repeated use, with efficiencies of 99.14%, 98.26%, and 96.35% recorded across three successive extraction/back-extraction cycles. These results highlight the IL system’s chemical robustness, low loss to the aqueous phase, and promising regeneration behavior, key attributes for scalable acid extraction processes.

### 2.2. Statistical Models

Based on Figure 1, Figure 2 and Figure 3, three levels (minimum, maximum, and mean) of each process factor were selected to obtain statistical models, as shown in Table 2. Statistical models described by Equation (1) link the predicted process responses (*E_j,pr_*, *j* = 1, 2) to *x*_1_, *x*_1_^2^, *x*_2_, *x*_2_^2^, *x*_3_, *x*_3_^2^, *x*_1_*x*_2_, *x*_1_*x*_3_, and *x*_2_*x*_3_, where *x*_1_, *x*_2_, and *x*_3_ are dimensionless process factors defined by Equations (2)–(4). Regression coefficients in Equation (1), i.e., *a*_0*j*_, *a*_1*j*_, *a*_11*j*_, *a*_2*j*_, *a*_22*j*_, *a*_3*j*_, *a*_33*j*_, *a*_12*j*_, *a*_13*j*_, and *a*_23*j*_, were identified from experimental values of process response variables (*E_j_*, *j* = 1, 2).

(1) $$E_{j,pr}=\alpha_{0j}+\alpha_{1j}x_{1}+\alpha_{11j}x_{1}^{2}+\alpha_{2j}x_{2}+\alpha_{22j}x_{2}^{2}+\alpha_{3j}x_{3}+\alpha_{33j}x_{3}^{2}+\alpha_{12j}x_{1}x_{2}+\alpha_{13j}x_{1}x_{3}+\alpha_{23j}x_{2}x_{3}$$

(2) $$x_{1}=\frac{pH-4}{1}$$

(3) $$x_{2}=\frac{IL-40}{40}$$

(4) $$x_{3}=\frac{t-35}{10}$$

Relevant statistics of *E_j_* and the values of regression coefficients, determination coefficient, *F* statistic, and *p*-value for *F*, which are summarized in Table 3, as well as the results of *t*-test for two samples assuming equal and unequal variances emphasize the following aspects:

1. *E*_1_ = *E_C_*_103_ ranged from 2.370% to 99.78% (72.58 ± 34.51%), and *E*_2_ = *E_C_*_104_ from 1.431% to 99.76% (75.05 ± 35.68%); *MN*_1_ = 72.58% and *MN*_2_ = 75.05% were not significantly different, i.e., *p* = 0.41 (one-tail) and *p* = 0.82 (two-tail);
2. Significant positive effects of dimensionless concentration of IL (*x*_2_), dimensionless temperature (*x*_3_), and *x*_3_^2^ as well as significant negative effects of dimensionless *pH* (*x*_1_), *x*_1_^2^, *x*_2_^2^, and *x*_2_*x*_3_ on *E*_1,*pr*_ = *E_C_*_103,*pr*_;
3. Significant positive effect of *x*_2_ and significant negative effects of *x*_1_, *x*_1_^2^, and *x*_2_^2^ on *E*_2,*pr*_ = *E_C_*_104,*pr*_;
4. A very good agreement between experimental and predicted values of process response variables (*R_j_*^2^ ≥ 0.9983, *R_j,adj_*^2^ ≥ 0.9974, *F_j_* ≥ 1117.4, and *p_j_* = 0.0000 for *j* = 1, 2).

**Figure 4:**
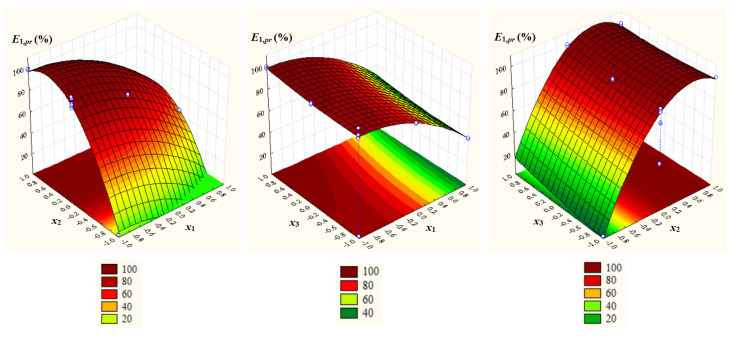
Surface and contour plots of *E*_1,*pr*_ = *E_C_*_103,*pr*_ depending on dimensionless process factors; *x*_1_ = *pH* − 4; *x*_2_ = (*IL* − 40)/40, *IL*: ILs concentration; *x*_3_ = (*t* − 35)/10, *t*: temperature.

**Figure 5:**
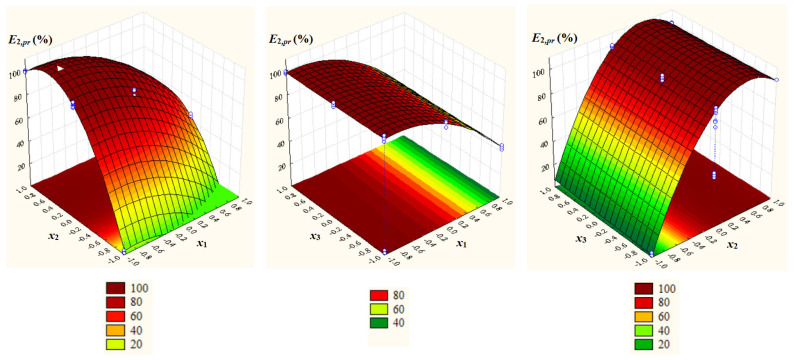
Surface and contour plots of *E*_2,*pr*_ = *E_C_*_104,*pr*_ depending on dimensionless process factors; *x*_1_ = *pH* − 4; *x*_2_ = (*IL* − 40)/40, *IL*: ILs concentration; *x*_3_ = (*t* − 35)/10, *t*: temperature.

Surface and contour plots of *E*_1,*pr*_ = *E_C_*_103,*pr*_ and *E*_2,*pr*_ = *E_C_*_104,*pr*_ depending on *x*_1_, *x*_2_, and *x*_3_ (Figure 4 and Figure 5) highlight a negative effect of *x*_1_ and a positive effect of *x*_2_ on both response variables, as well as a slightly positive effect of *x*_3_ on *E*_1,*pr*_ and a negligible effect of *x*_3_ on *E*_2,*pr*_.

### 2.3. Optimization of Process Factors

Optimization of extraction process factors, which was based on the desirability function, led to the optimal levels of process factors (*x*_1,*opt*_, *x*_2,*opt*_, and *x*_1,*opt*_, corresponding to *pH_opt_*, *IL_opt_*, and *t_opt_*) and predicted response variables (*E_j_*_,*pr*,*opt*_, *j* = 1, 2) specified in Table 3. To validate the statistical models defined by Equation (1), 3 experiments were performed at optimal levels of process factors, and related mean values of experimental response variables (*E_j_*_,*m*,*opt*_, *j* = 1, 2) and standard deviations (*SD_j_*) are summarized in Table 4.

The results of *t*-test for two samples with equal and unequal variances (*p_j_* ≥ 0.05) indicated that *E_j_*_,*m*,*opt*_ and *E_j_*_,*pr*,*opt*_ were not significantly different, which proves the validity of statistical models described by Equation (1). Moreover, *E*_2,*m*,*opt*_ = 99.24% was significantly higher than *E*_1,*m*,*opt*_ = 98.61%, i.e., *p* = 0.0067 (one-tail) and *p* = 0.0135 (two-tail).

## 3. Discussion

In reactive liquid–liquid extraction processes, VA is transferred from an aqueous phase into an organic phase, where unlike conventional solvent extraction, a chemical reaction occurs between the solute and the IL extractant. This interaction typically results in the formation of complexes in which VA binds to the ILs through specific interactions: hydrogen bonding, ion-pair formation, or a combination of both, depending on the nature of the extractant and the diluent. The formation of these complexes significantly influences extraction yield, alters the physical properties of the organic phase, and impacts the feasibility of its regeneration. Selecting an effective combination of ILs and solvent for the organic phase is crucial to achieving optimal performance in the extraction of carboxylic acids. In this context, ILs have emerged as highly promising components, serving both as extractants and as solvents, due to their unique physicochemical properties (low volatility and negligible vapor pressure), which contribute to their stability under a wide range of operating conditions. They are also characterized by broad solubility, and miscibility with various compounds, as well as relatively low toxicity compared to conventional organic solvents. These attributes position ILs as safer and more environmentally friendly alternatives for liquid–liquid extraction processes [29].

To optimize performance and reduce costs, conventional organic solvents can be employed as diluents, helping to modify the physical properties of the organic phase, such as lowering viscosity, which can improve the overall efficiency of the extraction process. Diluents can also influence the extraction depending on their nature. For example, active compounds like hexanol can stabilize extractant-acid complexes through hydrogen bonding, while inactive compounds such as hexane and dodecane do not participate in complex formation. In this study, heptane is selected as a diluent due to its ability to effectively reduce the viscosity of phosphonium-based ILs, thereby improving mass transfer during VA extraction, while maintaining phase stability and minimizing acid solubility in the diluent phase. Its chemical inertness and ease of recovery further support its suitability for efficient and sustainable IL-based extraction systems. ILs have proven effective in the recovery and separation of metals and organic/inorganic acid extraction, significantly enhancing separation efficiency and overall process performance, making them ideal candidates for efficient recovery [27,29].

**Figure 6:**
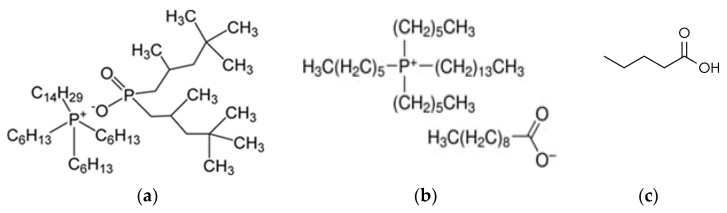
Chemical structure of ILs (**a**) C103 (trihexyl-tetradecylphosphonium bis(2,4,4-trimethylpentyl)phosphinate); (**b**) C104 (trihexyl-tetradecylphosphonium decanoate)); and (**c**) valeric acid.

The efficiency of carboxylic acid extraction is strongly influenced by pH, with optimal performance occurring under acidic conditions where the acid remains non-dissociated. The results from Figure 1 suggests that intermolecular interactions, such as hydrogen bonding, play a crucial role in VA extraction using ILs. Similar findings were reported by Reyhanitash et al. (2019), who emphasized that maintaining the *pH* below the *pK_a_* of VFAs is critical for an efficient extraction, as higher *pH* levels reduce the fraction of undissociated acids, essential for an effective separation [23]. However, the extraction process is also influenced by other factors, including functional group characteristics and steric hindrance. The extraction performance of phosphonium-based ILs is strongly influenced by the nature of the anion, particularly its basicity and ability to interact with carboxylic acids. In this study, C104 (trihexyl-tetradecylphosphonium decanoate) exhibited superior VA extraction efficiency compared to C103 (trihexyl-tetradecylphosphonium bis(2,4,4-trimethylpentyl)phosphinate), despite both sharing an identical cation (Figure 6). Hydrogen bonding occurs between the carboxylic acid group (–COOH) of valeric acid, acting as the hydrogen bond donor, and the anionic moieties of the ILs: in the case of C103, the interaction involves the carboxylate oxygen of the decanoate anion, whereas in C104, the phosphoryl oxygen (P=O) of the phosphinate anion serves as the hydrogen bond acceptor. 

This enhanced performance can be attributed to the higher basicity of the decanoate anion, whose conjugate acid has a pKa of approximately 4.9, compared to the reduced basicity and sterically hindered phosphinate anion. The increased basicity of decanoate promotes stronger acid-anion interactions, through hydrogen bonding thereby enhancing the partitioning of VA into the IL-rich phase. While the phosphinate anion contributes to hydrophobicity and may improve phase separation, its reduced proton affinity appears to limit effective acid binding. These findings underscore the importance of balancing hydrophobicity with anion basicity when designing IL systems for efficient extraction of organic acids.

To further elucidate the extraction mechanism, the effect of IL concentration on extraction efficiency was analyzed and used to estimate the stoichiometry of the resulting complexes. The loading ratio (*Z =* [*VA*]*_org_*/[*IL*]*_org_*) is a key parameter in reactive extraction processes, as it quantifies the extent to which the organic phase is loaded with an acid. It is typically defined as the ratio of the total concentration of acid in the organic phase (including undissociated, dimeric, and complexed forms) to the total extractant concentration in the same phase, effectively representing the amount of acid extracted per unit of extractant [32]. The value of *Z* helps in determining the type of complex formed between the acid and the extractant, which in turn is crucial for calculating the equilibrium complexation constant [33]:

1. At low *Z* values (*Z* < 0.5), a 1:1 acid-to-extractant complex is typically formed;
2. At intermediate *Z* values (0.5 < *Z* < 1), 2:1 or 1:2 acid-to-extractant complexes may form in the organic phase;
3. At high *Z* values (*Z* > 1), a 2:1 acid-to-extractant complex may form.

Thus, the loading ratio not only indicates the saturation level of the organic phase with acid but also plays a central role in characterizing the stoichiometry and strength of acid-extractant complexes during the extraction process [34,35].

The *Z* values for both Ils are presented in Table 5.

At lower levels of *IL* (0.031 and 0.06 mol/L for C103, respectively, 0.025 and 0.051 mol/L for C104), *Z* > 1. This indicates that the organic phase is heavily loaded with acid relative to the amount of extractant present, suggesting that more acid is being extracted per unit of extractant. Such conditions are typically associated with the formation of 2:1 acid-to-extractant complexes, as the extractant approaches or exceeds saturation and additional acid binds beyond the basic 1:1 stoichiometry. As the extractant concentration increases above the stoichiometric ratio (to 0.122 and 0.183 mol/L for C103, respectively, 0.103 and 0.155 mol/L for C104), 0.5 < *Z* < 1, reflecting a lower acid load per unit of extractant. This trend aligns with a dilution effect, where the same quantity of acid is shared among more extractant molecules, reducing the overall loading ratio. Under these conditions, particularly when the aqueous acid concentration remains constant, the formation of both 1:1 and 2:1 acid–IL complexes become possible.

Temperature plays a crucial role in the extraction efficiency of carboxylic acids using ILs. Moderate increases in temperature generally enhance efficiency by improving solubility and reducing the viscosity of the ILs. However, excessive heat may negatively affect extraction performance or lead to the degradation of the system. Identifying the optimal temperature is essential, as it depends on the specific carboxylic acid, the type of ILs used, and the presence of co-solvents. Temperature studies are particularly important not only for optimizing extraction conditions but also for ensuring compatibility with downstream processes such as back-extraction and solvent regeneration. Previous studies on carboxylic acids, including citric, lactic, succinic, acrylic, propionic, and butyric acids, have demonstrated that temperature, extractant and acid concentrations are key parameters affecting extraction performance [27,34]. The results for VA summarized in Table 6 highlight that both ILs showed increasing extraction efficiency, distribution coefficient (*K_d_*—defined as the ratio between VA concentration in organic and aqueous phase at equilibrium, [VA]*_org_*/[VA]*_aq_*) and extraction constant (*K_e_*—defined as [VA-IL]/[VA]^2^·[IL], for a 2:1 acid-to-IL complex, indicated by *Z* values higher that 1) with rising temperature up to 45 °C, indicating enhanced complexation and mass transfer at elevated temperatures. However, a notable decline in *K_d_* values was observed at 60 °C, suggesting that the complexes formation is exothermic, implicitly *E* could be affected by higher temperatures. Between the two extractants, C104 consistently outperformed C103, achieving higher *E* values at 25 °C, 35 °C, and 60 °C, due to its stronger hydrogen-bonding and solvating interactions with VA. The loading ratio (*Z*) remained relatively stable and below 2 for both ILs, supporting a 2:1 acid-to-extractant stoichiometry and indicating that extractant saturation was not reached.

The results of this study demonstrate that phosphonium-based Ils are effective extractants for valeric acid (VA), with extraction efficiency and complex stoichiometry strongly influenced by both pH and IL concentration, temperature having the smaller influence. These findings are consistent with previous studies on the extraction of short-chain carboxylic acids using ILs, where hydrogen bonding was identified as a key interaction mechanism [32,36]. The extraction efficiency of valeric acid using various extractant systems reported in the literature is summarized in Table 7. Compared to previous studies, the phosphonium-based ILs used in this work (C103 and C104) exhibit competitive or superior extraction efficiencies, reaching up to 99.24% under mild operating conditions.

The observed formation of 2:1 and 1:1 acid–extractant complexes aligns with reported behavior for similar systems, such as lactic and butyric acids, further supporting the hypothesis that ILs facilitate reactive extraction through specific molecular interactions rather than simple partitioning. C104 has been used for the separation of lactic acid from aqueous solutions, where the dominant complex is 2:1 lactic acid–IL species in the aqueous acid concentration range of 0.2 to 2.0 mol/L. At higher concentrations, the formation of a 3:1 complex also becomes significant, while the 1:1 complex is primarily formed at lactic acid concentrations below 0.2 mol/L [36]. Importantly, the ability to modulate complex formation through extractant concentration highlights the flexibility of IL-based systems and their potential for process optimization. From a broader perspective, these results contribute to the growing evidence supporting ILs as greener and more efficient alternatives for carboxylic acid recovery. Future research should explore the extraction behavior under continuous operation or in the presence of mixed acid systems, to assess their feasibility for industrial-scale applications.

## 4. Materials and Methods

### 4.1. Chemicals and Procedures

All chemicals, including VA (97.0%), C103—trihexyl-tetra-decyl-phosphonium bis(2,4,4-trimethylpentyl)phosphinate (95%), C104—trihexyl-tetra-decyl-phosphonium decanoate (95%), heptane (99%), methanesulfonic acid (99%), sodium hydroxide (>97%), sulfuric acid (95.0–98.0%), and acetonitrile (99.99%), were purchased from Merck (Merck KGaA, Darmstadt, Germany) and used as received. The VA extraction experiments were conducted for 10 min, at 25–65 °C, using a vibration shaker (WIZARD IR Infrared Vortex Mixer, VELP Scientifica Srl, Usmate (MB), Italy) with a stirring speed of 1200 rpm. Equal volumes (2 mL) of VA solution and organic phase were mixed in a glass cell, with initial concentration of VA in the aqueous phase of 10 g/L. Extraction was carried out using two hydrophobic ILs, i.e., either [C_14_C_6_C_6_C_6_P][Dec]—CYPHOS IL 103 (C103) or [C_14_C_6_C_6_C_6_P][(iOc)_2_Phos]—CYPHOS IL 104 (C104), mixed with *n*-heptane. The concentration of ILs in the organic phase ranged from 0 to 120 g/L. The pH of the initial aqueous phase was adjusted to the predetermined values (3, 4, 5, or 6) using 4% sulfuric acid and 4% sodium hydroxide solutions, based on readings from a Hanna Instruments pH 213 digital pH meter (Woonsocket, Rhode Island). The pH of the VA solution (10 g/L) was 3.05 before any adjustments. After extraction, the samples were separated by centrifugation (DLAB centrifuge (Beijing, China) at 4000 rpm for 5 min. Stripping experiments for the recovery of valeric acid (VA) were conducted using a diluted sodium hydroxide solution at pH 12, in equal volume (2 mL) to the VA-loaded organic phase. The two phases were contacted in a vibratory shaker at 1200 rpm for 20 min. Following the extraction step, the exhausted aqueous phase was removed, and the loaded organic phase was subsequently mixed with the stripping solution to facilitate back-extraction of VA. All experiments were performed in triplicate (*n* = 3). Process analysis was carried out by calculating the extraction efficiency, *E* (%), using VA concentrations in the initial, raffinate and stripping solutions. A Dionex Ultimate HPLC system (Thermo Fisher Scientific Inc., Waltham, MA, USA), equipped with an Acclaim OA column (temperature 30 °C), was used for analysis. The mobile phase consisted of 0-1 min with 2.5 mM methanesulfonic acid, followed by 1–11 min with a mixture of 45% acetonitrile and 55% methanesulfonic acid solution, with a flow rate of 0.6 mL/min and detection at 210 nm.

### 4.2. Statistical Analysis, Modeling, and Factor Optimization

One-way ANOVA was used to determine that the effects of process quantitative factors (aqueous phase pH, IL concentration, and extraction temperature) and extractant type (C103 and C104) on extraction efficiency (*E*) were significant (*p* < 0.05) or not (*p* ≥ 0.05). Student *t*-test for two samples with equal and unequal variances was applied to assess if the predicted and mean experimental values of *E_C_*_103_ and *E_C_*_104_ under optimal conditions were significantly different or not. For each IL, the effects of process factors on *E* were quantified using a response surface regression model, and the process factors were optimized based on the desirability function approach [41].

## 5. Conclusions

In conclusion, the reactive extraction of VA using phosphonium-based ILs in a heptane diluent has proven to be highly effective. Among the extractants tested, trihexyl-tetra-decyl-phosphonium bis(2,4,4-trimethylpentyl)phosphinate (C104) outperformed trihexyl-tetra-decyl-phosphonium decanoate (C103), exhibiting superior distribution ratios and extraction efficiencies. The extraction mechanism involves acid–IL complex formation through hydrogen bonding, with observed stoichiometries of 1:1 and 2:1 depending on the extractant concentration. Optimal extraction conditions, i.e., pH of 3.8 and 4, respectively, an IL concentration of 60 g/L, and temperature of 25 °C, resulted in optimal mean values of extraction efficiencies of 98.61% and 99.24% for C103 and C104, respectively. These findings highlight the potential of phosphonium ILs for the efficient recovery of VA from dilute aqueous waste streams and fermentation broths, offering a promising route for process development in biorefinery applications.

## Figures and Tables

**Table 1:** Comparison of VA yields in waste sludge fermentation processes.

Pretreatment	VA (mg COD/g VSS)	VA (% COD)	VFA Production (mg COD/g VSS)	Reference
In situ ammonia stripping	77.2	25	308	[15]
Sulfite	16	4.9	324	[16]
Riboflavin	220.1	62.8	355	[6]
Saponin	113.8	40	292	[17]
Tetrakis hydroxymethyl phosphonium sulfate	72.7	37.5	194	[18]

COD—chemical oxygen demand; VA—valeric acid; VFA—volatile fatty acid; VSS—volatile suspended solids.

**Table 2:** Levels of dimensional and dimensionless factors.

No.	*X*_1_ = *pH*	*X*_2_ = *IL* (g/L)	*X*_3_ = *t* (°C)	*x*_1_	*x*_2_	*x*_3_
1	3	40	25	−1	0	−1
2	4	40	25	0	0	−1
3	5	40	25	1	0	−1
4	3	0	25	−1	−1	−1
5	3	80	25	−1	1	−1
6	3	40	35	−1	0	0
7	3	40	45	−1	0	1
*i*	1	2	3	1	2	3
*MIN_i_*	3	0	25	−1	−1	−1
*MAX_i_*	5	80	45	1	1	1
*MN_i_*	4	40	35	0	0	0

*MIN*, *MAX*, and *MN*—minimum, maximum, and mean values; *x_i_* = (*X_i_* − *MN_i_*)/(*MAX_i_* − *MN_i_*), *i* = 1…3.

**Table 3:** Relevant characteristics of extraction response variables.

Experimental Extraction Efficiency	*E_j_* (%)	
*j*	1 (C103)	2 (C104)
Minimum value, *MIN_j_*	2.370	1.431
Maximum value, *MAX_j_*	99.78	99.76
Mean value, *MN_j_*	72.58	75.05
Standard deviation, *SD_j_*	34.51	35.68
Coefficient of variation, *CV_j_* = 100*SD_j_*/*MN_j_*	47.55	47.54
Predicted extraction efficiency	*E_j,pr_* (%)	
Regression coefficients		
*α*_0*j*_	**82.111**	**87.155**
*α*_1*j*_	**−23.851**	**−26.500**
*α*_11*j*_	**−13.077**	**−16.166**
*α*_2*j*_	**44.444**	**47.202**
*α*_22*j*_	**−40.515**	**−45.790**
*α*_3*j*_	**3.6882**	0.8649
*α*_33*j*_	**2.0150**	0.3300
*α*_12*j*_	0.0000	0.0000
*α*_13*j*_	0.0000	0.0000
*α*_23*j*_	**−3.6882**	−1.3974
Coefficient of determination, adjusted coefficient of determination, *F* statistic and its *p_j_*-value		
*R_j_* ^2^	0.9994	0.9983
*R_j,adj_* ^2^	0.9991	0.9974
*F_j_*	3264.6	1117.4
*p_j_*	0.0000	0.0000

Significant regression coefficients are highlighted in bold.

**Table 4:** Predicted and experimental values of process response variables at optimal levels of dimensionless and dimensional factors.

*j*	*x*_1,*opt*_	*x*_2,*opt*_	*x*_3,*opt*_	*pH*_*opt*_	*IL*_*opt*_ (g/L)	*t*_*opt*_ (°C)	*E*_*j,pr,opt*_ (%)	*E*_*j,m,opt*_ ± *SD*_*j*_ (%)
1	−0.2	0.5	−1	3.8	60	25	98.62	98.61 ± 0.18
2	0	0.5	−1	4	60	25	99.47	±0.19

**Table 5:** Loading factor values for VA extraction depending on *IL* (initial VA concentration: 10 g/L = 0.098 mol/L).

Extractant	C103		C104	
*IL* (g/L)	*IL* (mol/L)	*Z*	*IL* (mol/L)	*Z*
20	0.031	1.88	0.025	2.61
40	0.061	1.46	0.051	1.83
80	0.122	0.79	0.103	0.94
120	0.183	0.53	0.155	0.63

*IL*—initial concentration of ILs; *Z*—loading ratio.

**Table 6:** Temperature influence on VA extraction (initial VA concentration: 10 g/L, *IL* = 40 g/L, *pH* = 3).

Extractant	C103				C104			
*t* (°C)	*E_m_* (%)	*K_d_*	*Z*	*K_e_*	*E_m_* (%)	*K_d_*	*Z*	*K_e_*
25	91.21	10.18	1.57	70.74	96.95	31.31	1.83	532.93
35	92.89	13.06	1.58	114.05	97.49	38.82	1.85	814.26
45	98.59	73.33	1.60	3388.06	98.68	74.97	1.87	3000.17
60	90.05	9.22	1.57	58.64	94.23	16.01	1.78	143.48

*IL*—ILs concentration; *t*—temperature; *E_m_*—mean value of extraction efficiency; *K_d_*—distribution coefficient; *K****_e_***—extraction constant; *Z*—loading ratio.

**Table 7:** Comparative extraction efficiency of VA using various extractant systems [37,38,39,40].

Study	Extractant System	Valeric Acid Concentration (mol/L)	Max. Extraction Efficiency (%)
Baylan (2019) [37]	[HMIM][PF_6_] + TBP	0.10–0.30	87.96
Firdous & Ahmad (2020) [38]	TBP + kerosene	0.05–0.13	97.64
Senol (2015) [39]	TPA + ethyl valerate	0.10	99.07
Mukherjee & Munshi (2022) [40]	40% TBP + sunflower oil	0.01–0.10	96.42
	40% TBP + soybean oil	0.01–0.10	96.18
This work	C103/C104 + heptane	0.097	98.61/99.24

[HMIM][PF_6_]—1-hexyl-3-methylimidazolium hexafluorophosphate; TBP—tributyl phosphate; TPA—tripropylamine.

## Data Availability

The original contributions presented in this study are included in the article. Further inquiries can be directed to the corresponding authors.

## Abbreviations

The following abbreviations are used in this manuscript:

C103	trihexyl(tetradecyl)phosphonium decanoate
C104	trihexyl(tetradecyl)phosphonium bis(2,4,4-trimethylpentyl)phosphinate
VA	valeric acid
IL	ionic liquid
